# m5C RNA Methylation Regulators Predict Prognosis and Regulate the Immune Microenvironment in Lung Squamous Cell Carcinoma

**DOI:** 10.3389/fonc.2021.657466

**Published:** 2021-06-09

**Authors:** Junfan Pan, Zhidong Huang, Yiquan Xu

**Affiliations:** ^1^ Department of Thoracic Oncology, Fujian Medical University Cancer Hospital, Fujian Cancer Hospital, Fuzhou, China; ^2^ Shengli Clinical Medical College of Fujian Medical University, Fuzhou, China; ^3^ Quanzhou First Hospital of Fujian Medical University, Quanzhou, China

**Keywords:** RNA methylation, tumor, lung squamous cell carcinoma, 5-methylcytosine, prognosis, tumor immune microenvironment

## Abstract

RNA methylation is a novel epigenetic modification that can be used to evaluate tumor prognosis. However, the underlying mechanisms are unclear. This study aimed to investigate the genetic characteristics of 5-methylcytosine (m5C) and N1-methyladenosine (m1A) regulators in lung squamous cell carcinoma (LUSC) and the prognostic value and immune-related effects of m5C regulators. To this end, we selected the public LUSC dataset from the Cancer Genome Atlas and Gene Expression Omnibus. The least absolute shrinkage and selection operator regression model was used to identify prognostic risk signatures. We used the UALCAN and Human Protein Atlas databases to study the expression of target gene mRNA/protein expression. Furthermore, the Tumor Immune Single Cell Hub and the Tumor Immune Estimation Resource were used to evaluate the degree of immune cell infiltration. Most of the m5C and m1A regulators showed significantly different expression between LUSC and normal samples. The m5C regulators were associated with poor prognosis. In addition, a prognostic risk signature was developed based on two m5C regulators, NOP2/Sun RNA methyltransferase 3 (*NSUN3*), and NOP2/Sun RNA methyltransferase 4 (*NSUN4*). Compared with normal lung tissues, the expression of *NSUN3* and *NSUN4* in the LUSC TCGA dataset was increased, which was related to clinicopathological characteristics and survival. *NSUN3* and *NSUN4* were related to the infiltration of six major immune cells; especially *NSUN3*, which was closely related to CD8+ T cells, while *NSUN4* was closely related to neutrophils. Our findings suggest that m5C regulators can predict the clinical prognosis risk and regulate the tumor immune microenvironment in LUSC.

## Introduction

Lung cancer is the leading cause of cancer-related deaths worldwide. Each year, 1.8 million people are diagnosed with lung cancer, and 1.6 million people die from the disease (1, 2). Approximately 85% of lung cancer patients have the non-small cell lung cancer (NSCLC) subtype (3). More than half of the patients diagnosed with lung cancer die within one year after diagnosis, and the 5-year survival rate is approximately 17.8%. NSCLC includes three types: adenocarcinoma, squamous cell carcinoma, and large cell carcinoma. Lung squamous cell carcinoma (LUSC), which accounts for about 40% of NSCLC, is closely related to smoking and economic levels (4). Compared with lung adenocarcinoma (LUAD), LUSC has a poor clinical prognosis and lacks targeted drugs. Therefore, a more comprehensive understanding of the molecular mechanism of LUSC progression is essential for the development of new treatment methods.

Known mRNA post-transcriptional modifications include 5-terminal capping, pre-mRNA splicing, polyadenylation, and mRNA export epigenetic mechanisms (5). RNA modification is also controlled by writers, erasers, and readers. Writers are the proteins that add chemical modification to specific sites of RNA molecules, erasers remove chemical modification added by the writers, and readers can recognize and bind RNA modification sites (6). These proteins work together as a complex network that dynamically regulates RNA modification. The internal modifications of mRNA mainly include N6-methyladenosine (m6A), N1-methyladenosine (m1A), 5-methylcytosine (m5C), 5-hydroxymethyl cytosine (hm5C), N7-methylguanosine (m7G), and pseudopurine (C) (7). m6A is the most common post-transcriptional modification method and is enriched in many eukaryotes and prokaryotes. With the application of high-throughput sequencing technology, it was found that m6A was mainly distributed in the coding region, and 30 untranslated regions were significantly enriched upstream of the stop codon (8). Increasing numbers of studies have shown that changes in m6A affected tumor progression, including proliferation, growth, invasion, and metastasis (9). In addition, studies have shown that METTL3-mediated m6A mRNA methylation improved the stability of *YAP* mRNA by regulating the MALAT1-miR-1914-3p-YAP axis and increased the induction of NSCLC drug resistance and metastasis (10).

5-Methylcytosine (m5C) is a widespread mRNA modification discovered in 1925, located in the untranslated regions (UTRs) of mRNA transcripts (11). Previous studies have shown that m5C played an important regulatory role in many aspects of gene expression, including RNA export, ribosome assembly, and translation (12). A recent study has shown that m5C also played an important role under pathological conditions (13), such as in cancer; *NSUN2* and *YBX1* promoted pathogenesis in human bladder urothelial carcinoma by targeting the m5C methylation site in the untranslated region of *HDGF3*. Adding a methyl group to the N1 position of adenosine will form an m1A, which appears mainly upstream of the initiation codon of the first splicing site and has a strong enrichment effect on translation in the 5’UTR (14). However, little is known about the function of m5C and m1A regulators in NSCLC.

In recent years, a large number of studies have proved that the tumor immune microenvironment (TIM) played a vital role in cancer progression and therapeutic efficacy. Li et al. (15) found that there was no significant difference in lymphocyte infiltration between the low and high immune risk groups of non-squamous NSCLC in the Cancer Genome Atlas (TCGA) dataset. In the high-immune risk group, it was found that the level of neutrophil necrosis and infiltration was increased significantly. The inflammatory tumor microenvironment is known to be associated with a poor prognosis. Liu et al. (16) found that in the early clinical stage of LUAD, lack of memory B cells or increased M0 macrophages were related to poor prognosis. In LUSC, T follicle helper cells were associated with good prognosis, while an increase in the number of neutrophils indicated poor prognosis. Previous studies have shown that mRNA post-transcriptional modification was associated with the progression and prognosis of LUSC. However, the relationship between mRNA post-transcriptional modification and the TIM in LUSC remains unclear.

In this study, we used the TCGA database and GEO dataset to conduct an in-depth analysis of m5C and m1A regulators in LUSC tissues and adjacent normal tissues. The purpose of this study was to explore the regulation of m5C and m1A in LUSC. Specifically, differentially expressed genes, clinicopathological characteristics, differences in survival, and impact on the TIM were addressed to provide therapeutic significance for the treatment of LUSC.

## Materials and Methods

### Data Acquisition

Transcriptome analysis of raw data and corresponding clinical information of the LUSC cohort were downloaded from TCGA data portal (http://cancergenome.nih.gov/). A total of 551 research samples were obtained, including 502 LUSC tissues and 49 normal lung tissues, as was the corresponding clinical information (**Table 1**), as the training cohort. Independent gene microarray data from Gene Expression Omnibus (GEO) public datasets (https://www.ncbi.nlm.nih.gov/geo/) served as a validation cohort. Three datasets, GSE3349, GSE3141, and GSE19188, were selected, with a total of 125 samples including 82 LUSC tissues and 43 normal lung tissues.

**Table 1:** The clinical characteristics of lung squamous cell carcinoma patients in the training cohort.

Variables	No. of Patients	Percentage (%)
Age (years)		
≤65	189	38.3
>65	303	61.5
Unknown	1	0.2
Gender		
Male	364	73.8
Female	129	26.2
T stage		
T1	110	22.3
T2	289	58.6
T3	70	14.2
T4	24	4.9
N stage		
N0	313	63.5
N1	129	26.2
N2	40	8.1
N3	5	1.0
Unknown	6	1.2
M stage		
M0	405	82.2
M1	7	1.4
Unknown	81	16.4
Pathological stage		
I	240	48.7
II	158	32.1
III	84	17.0
IV	7	1.4
Unknown	4	0.8
Total	493	100.0

### Selection of Differentially Expressed Genes in the TCGA Database

A total of 9 m1A regulators were obtained from the published literature, including *YTHDF2*, *RRP8*, *ALKBH3*, *YTHDC1*, *TRMT61A*, *YTHDF1*, *ALKBH1*, *TRMT6*, and *YTHDF3*; there were 15 m5C regulators, including *NSUN1*, *NSUN*, *NSUN3*, *NSUN4*, *NSUN5*, *NSUN6*, *NSUN7*, *ALYREF*, *DNMT1*, *DNMT2*, *DNMT3A*, *DNMT3B*, *TET2*, *TRDMT1*, and *YBX1* (**Table 2**). Among them, *NSUN1* and *DNMT2* were not found in the TCGA LUSC data. Extract of the expression matrix and clinical data of m1A and m5C regulators of 502 LUSC samples and 42 normal lung tissues were obtained from the TCGA database. Then, the R version (4.0.2) of the limma software package was used to identify the m1A regulators and m5C regulators that were differentially expressed between the tumor and the control groups. *P* values <0.05 and |log2(FC)|>1 were considered to indicate the significance threshold in all tests. In addition, we used heat maps and violin maps to visually show the differential expression of m5C regulators and m1A regulators between the two groups.

**Table 2:** The list of the RNA modifying proteins involve in m1A, m5C.

Regulators	Type
m1A	
TRMT6	“writers”
TRMT61A	“writers”
RRP8	“writers”
ALKBH1	“readers”
ALKBH3	“readers”
YTHDF1	“erasers”
YTHDF2	“erasers”
YTHDF3	“erasers”
YTHDC1	“erasers”
m5C	
TRDMT1	“writers”
NSUN1	“writers”
NSUN2	“writers”
NSUN3	“writers”
NSUN4	“writers”
NSUN5	“writers”
NSUN6	“writers”
NSUN7	“writers”
DNMT1	“writers”
DNMT2	“writers”
DNMT3A	“writers”
DNMT3B	“writers”
ALYREF	“readers”
YBX1	“erasers”
TET2	“erasers”

### GEO Database Verified Differentially Expressed Genes

We first integrated all of the samples in the two datasets, using the sva package in the R computing environment for batch normalization, which increased the number of samples and avoided unreliable results (a total of 72 samples, including 29 LUSC samples and 43 healthy controls). Next, we performed differential analysis (|Log2FC|>2, adjusted *p*-value <0.05) by comparing tumor tissues to normal tissues in the R computing environment using the limma package. Subsequently, heat maps and violin maps were used to visually show the expression differences between the two groups.

### Construction of the Protein–Protein Interactions Network

To further screen out the hub genes, we use the Search Tool for the Retrieval of Interacting Genes (STRING) database (https://string-db.org/) to analyze the differentially expressed m5C regulators to construct the Protein–Protein Interactions (PPI) network (17). The threshold of PPI network hub genes was the minimum gene interaction score >0.4. This network uses an evidence model to predict the association between proteins based on up to seven different types of evidence (fusion evidence, neighborhood evidence, co-occurrence evidence, experimental evidence, text mining evidence, database evidence, and co-expression evidence).

### Construction and Validation of the Prognostic Risk Scoring Model

To evaluate the prognostic value of the m5C regulators, we performed univariate Cox regression analysis (18).Then, we used the following formula to calculate the risk score of the prognostic characteristics in each patient: risk score = coefficient 1 ∗ value1 + coefficient 2 ∗ value 2, where coefficient was determined using the least absolute shrinkage and selection operator (LASSO) algorithm, and the value was the relative expression level of each selected gene. Finally, based on the median risk score, LUSC patients included in the TCGA database were stratified into high-risk and low-risk groups. Survival differences between the two risk groups were evaluated using the Kaplan–Meier (KM) survival curve. According to the risk score formula, we combined GSE3141 with GSE19188 as a validation set to verify the reliability of the risk score model.

### UALCAN Database

The UALCAN online database (http://ualcan.path.uab.edu) can analyze the correlation between gene expression and clinicopathological characteristics and survival according to different subtypes of the disease. Using the UALCAN database, we analyzed the database based on clinicopathological parameters such as age, race, tumor grade, smoking, and *TP53* mutations.

### Human Protein Atlas

Immunohistochemistry (IHC) showed a difference in the expression of *NSUN3* and *NSUN4* proteins in human normal lung and LUSC tissues from the Human Protein Atlas (HPA) website (https://www.proteinatlas.org). According to the staining intensity (negative, weak, medium, strong) and the proportion of staining cells (<25, 25–75%, or >75%), immunoreactivity score (IRS) was divided into four levels: 1) No detection; 2) Low; 3) Medium staining; 4) High staining.

### CBioPortal Database

We used the cBioPortal platform (http://www.cbioportal.org/) to analyze the prognostic-related m5C regulators changes in LUSC (including missense mutations, fusion, amplification, and deep deletion) in TCGA. All searches were performed according to the online instructions at the cBioPortal.

### Gene Enrichment Analysis

Gene Enrichment Analysis (GSEA) was performed in the LUSC cohort to gain insight into the biological pathways of the high-risk and low-risk subgroups defined by 13 gene expression characteristics. GSEA was used to find rich terms predicted to be associated with the Kyoto Encyclopedia of Genes and Genomes (KEGG) pathway in C2. Terms enriched in hub genes with p <0.01 and FDR (Error Found Rate) <0.05 were considered statistically significant.

### Tumor Immune Single Cell Hub Database

The Tumor Immune Single-Cell Hub (TISCH) (http://tisch.comp-genomics.org) was a RNA-seq database focused on the tumor microenvironment (TME). TISCH provided detailed cell type annotations at the single-cell level, allowing TME to explore across different cancer types (17). In this study, the TISCH database was used to analyze the heterogeneity of TME in different datasets and different cells.

### TIMER Database

The TIMER database (https://cistrome.shinyapps.io/timer/) was used to evaluate the potential correlation between prognostic-related m5C regulators and tumor infiltrating lymphocytes. It can use the immune penetration algorithm to calculate the infiltration abundance of the six immune cells (B cells, CD4+ T cells, CD8+ T cells, neutrophils, macrophages, and dendritic cells) in the TCGA database. In addition, it provides three main analysis modules: Immune, Exploration, and Estimation. The Immune module includes clinical outcomes, somatic mutation, and somatic copy number change, enabling users to comprehensively analyze the relationship between immune cell infiltration and multiple factors (19).

### Statistical Analysis

One-way analysis of variance was used to compare the expression levels of 13 m5C regulators and nine m1A regulators in 502 LUSC tissues and 49 normal lung tissues. The Spearman test was used to identify the correlation between m5C regulators. The median risk value was used as a cut-off value to divide patients into high- and low-risk groups. Kaplan–Meier method was used to assess the correlation between high- and low-risk groups and survival. Univariate and multivariate COX regression analyses identified whether risk score, gender, race, grade, smoking, *TP53* mutation, *etc.* can be used as independent prognostic factors. All analyses used R v3.6.0 and SPSS v25.0 software. *p* values <0.05 were considered statistically different.

## Results

### The Differentially Expressed m5C and m1A Regulators Between LUSC and Normal Control Samples

**Figure 1:**
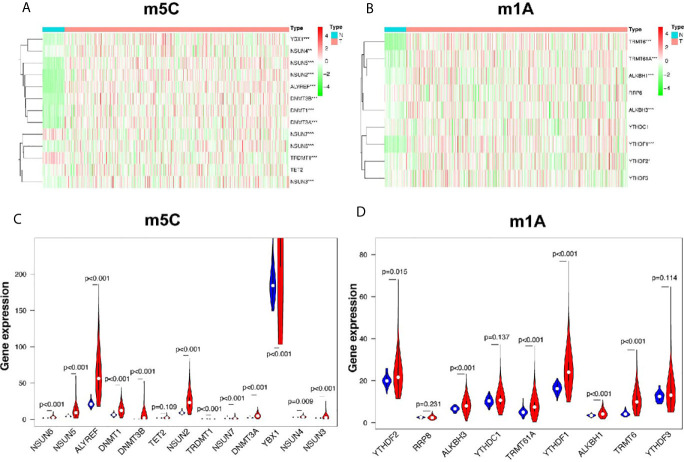
Identification of differential genes between m5C and m1A regulators in LUSC and normal groups. **(A, C)** The heatmap and violin plot visually show the expression differences in m5C regulators between the two groups. (In the tumor group, the expression interval of *YBX1* was large, and only partial data were intercepted.) **(B, D)** The heatmap and violin plot visually show the expression differences in m1A regulators between the two groups. N, normal samples; T, tumor samples; blue violins represent normal samples; red violins represent tumor samples; *p < 0.05, **p < 0.01, ***p < 0.001.

In this study, 502 LUSC tissues and 49 normal tissues from TCGA were analyzed. The results showed that most of the m5C regulators were differently expressed between LUSC and normal tissues (**Figure 1A**). Nine genes, including *NSUN6* (*p* < 0.001), *NSUN5* (*p* < 0.001), *ALYREF* (*p* < 0.001), *DNMT1* (*p* < 0.001), *DNMT3B* (*p* < 0.001), *NSUN2* (*p* < 0.001), *DNMT3A* (*p* < 0.001), *YBX1* (*p* < 0.001), and *NSUN3* (*p* < 0.001) were significantly up-regulated in LUSC samples (*p* < 0.001) compared to those in normal tissues. In addition, the expression levels of two genes, *TRDMT1* and *NSUN7*, were significantly down-regulated in LUSC tissues (both p < 0.001) compared to those in normal tissues. However, the levels of *TET2* and *NSUN4* were not significantly different (**Figure 1C** and **Supplementary Table 1**). The expression of the m1A regulators also differed between LUSC tissues and adjacent normal tissues (**Figure 1B**). *ALKBH3* (*p* < 0.001), *TRMT61A* (*p* < 0.001), *YTHDF1* (*p* < 0.001), *ALKBH1* (*p* < 0.001), and *TRMT6* (*p* < 0.001) in cancer tissues were significantly up-regulated compared to those in normal tissues (*p* < 0.001). The expression of *YTHDF2* in cancer tissues was also up-regulated (*p* < 0.05), while those of *RRP8*, *YTHDC1*, and *YTHDF3* were not significantly different (**Figure 1D** and **Supplementary Table 1**).

### Correlation Between m5C, m1A Regulators, and Overall Survival in LUSC Patients

**Figure 2:**
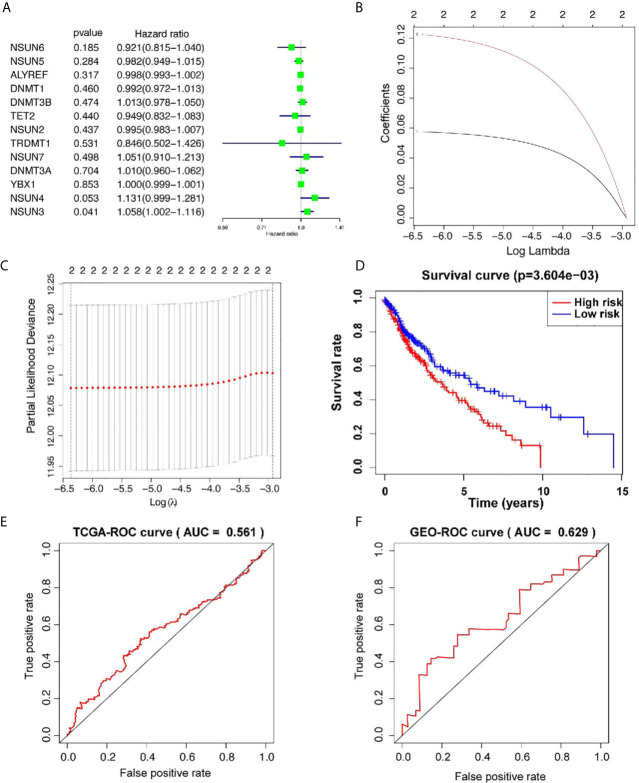
Prognostic risk model construction. **(A)** Univariate Cox regression analysis indicates that *DNMT3B*, *NSUN7*, *DNMT3A*, *NSUN3*, and *NSUN4* are the risk genes for LUSC with the hazard ratio (HR) >1. **(B, C)** The coefficients of genes are obtained using least absolute shrinkage and selection operator (LASSO) algorithm. Ten-fold cross-validation used for tuning parameter selection in the LASSO model **(D)** Kaplan–Meier curves of OS between the high risk and low risk groups. **(E)** The 3-year receiver operating characteristic (ROC) curves of the training cohort. **(F)** The 3-year receiver operating characteristic (ROC) curves of the validation cohort. AUC, area under ROC curve.

In order to study the correlation between m5C and m1A regulators and the prognosis of LUSC, we used univariate cox regression to analyze the relationship between m5C and m1A regulators and OS in the TCGA database. The results of the m5C regulators showed that *NSUN3* [hazard ratio (HR) = 1.057, 95% confidence interval (CI) = 1.002–1.115, *p* = 0.040] and *NSUN4* (HR = 1.130, 95% CI = 0.998–1.280, *p* = 0.052) were high risks, showing an HR of >1 (**Figure 2A**). Next, we used these two genes to build a prognostic risk model (**Figures 2B, C**). LASSO algorithm was used to calculate the correlation coefficient (**Table 3**). The risk value for each patient with LUSC was calculated as follows: risk score = 0.057 * NSUN3 + 0.122 * NSUN4. After that, according to the median risk value, LUSC patients in the TCGA database were divided into high-risk and low-risk groups, and further survival analysis was performed in these groups. As shown in **Figure 2D**, the OS in the high-risk group was significantly lower than that in the low-risk group (*p* < 0.001). In order to evaluate the accuracy of prognostic-related risk values for predicting prognosis, we conducted a time-dependent ROC analysis. The area under the ROC curve (AUC) for the 3-year analyses in the training datasets was 0.561 (**Figure 2E**). At the same time, the samples in the validation datasets (n = 85) were also divided into high-risk groups (n  = 42) and low-risk groups (n = 43) using the same method. In the validation set, AUC = 0.629 for the 3-year analyses (**Figure 2F**), indicating the risk score model can better predict the prognosis. The results of the m1A regulators showed that only *ALKBH1* was correlated with OS (HR = 0.865, 95% CI = 0.781–0.957, *p* = 0.005), while the rest had *p >*0.05, and *ALKBH1* was the protective factor of HR <1 (**Supplementary Figure 2**). Therefore, this study focused on the prognostic value of m5C regulators in LUSC.

**Table 3:** Genes selected to build risk signature and the corresponding coefficients.

Genes	Coefficients
NSUN3	0.0575621521710856
NSUN4	0.122380087085298

### GEO Database Verified Differentially Expressed Genes in m5C Regulators

**Figure 3:**
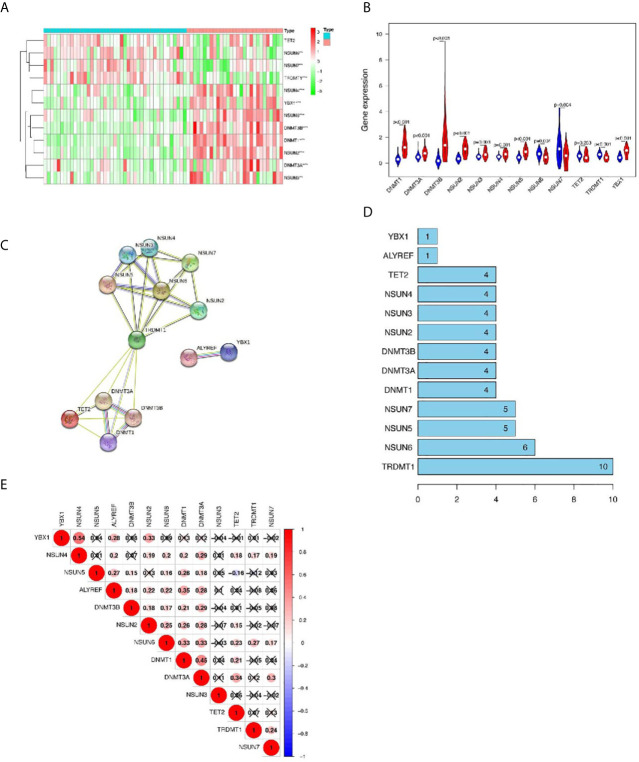
The GEO database verifies the differentially expressed genes (DEGs) in m5C regulators and the interaction and correlation between m5C regulators. **(A, B)** Comparison of m5C regulators between tumor and normal groups in GEO datasets. **(C, D)** The protein–protein interaction (PPI) network shows the interaction between differentially expressed genes among m5C regulators. **(E)** The Pearson correlation analysis of the m5C regulators. *p < 0.05,**p < 0.01,***p < 0.001.

Next, we used the GEO database to further verify differentially expressed genes in the m5C regulators. Compared with single array analysis, the integration of multiple arrays can improve the reliability of the results. Therefore, we first integrated all the samples from the two datasets to increase the sample size. The results showed that there were significant differences in the expression of m5C regulators between LUSC and normal tissues (**Figure 3A**). Among them, the expression levels of *NSUN5*, *DNMT1*, *DNMT3B*, *NSUN2*, *DNMT3A*, *YBX1*, *NSUN3*, and *NSUN4* in cancer tissues were significantly higher than those in normal tissues (p < 0.001); the expression levels of *NSUN6*, *TRDMT1*, and *NSUN7* in cancer tissues were significantly lower than those in normal tissues (p < 0.001). No significant difference was found in the expression levels of *TET2* between the two groups; *ALYREF*, *DNMT2*, and *NSUN1* were not found in the database (**Figure 3B** and **Supplementary Table 2**).

### Interaction and Correlation Between m5C Regulators

Next, we further analyzed the interaction between the m5C regulators. As shown in **Figures 3C, D**, *TRDMT1* was the hub gene of the network and interacts with 10 other genes. In the correlation analysis, *TRDMT1* did not show a strong correlation with other genes. However, interestingly, *ALYREF*, *NSUN2*, *NSUN6*, *DNMT1*, and *DNMT3A* had weak to moderate correlations with other genes. *NSUN4* and *YBXI* had the strongest correlation (**Figure 3E**). The above results indicated that there was a certain interaction between m5C regulators.

### Prognosis-Related Risk Values in LUSC Were Not Only Related to Clinical Outcome and Clinicopathological Characteristics, but Also an Independent Prognostic Factor in LUSC

**Figure 4:**
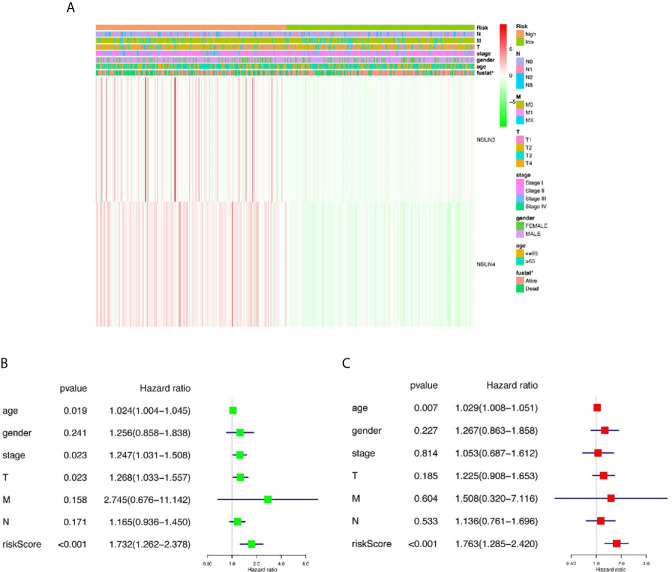
The relationship between risk score and clinical outcome, pathological characteristics, and prognostic value of LUSC. **(A)** Expression differences in clinicopathological characteristics and risk scores between high and low risk groups from The Cancer genome Atlas (TCGA) dataset. **(B)** Univariate Cox regression analysis of clinicopathological characteristics and risk score. **(C)** Multivariate Cox regression analysis of clinicopathological characteristics and risk score. *p < 0.05, **p < 0.01, ***p < 0.001.

Then, we further analyze the risk value and clinicopathological characteristics. **Figure 4A** indicated that the LUSC patients in the high-risk group generally contained a higher proportion of *NSUN3* and *NSUN4* than those in the low-risk group. In addition, significant differences in terms of survival state (*p* < 0.05) were also observed between the high-and low-risk groups. However, no significant differences were found between the two groups for gender, T, N, M, pathological stage, and age. Next, we used univariate Cox and multivariate Cox regression to analyze whether the prognostic risk values in LUSC can be used as an independent prognostic factor. Univariate Cox regression analysis showed that age (HR = 1.024, 95% CI =1.003–1.044, *p* = 0.019), pathological stage (HR = 1.024, 95% CI =1.003–1.044, *p* = 0.019), T (HR =1.267, 95% CI =1.032–1.556, *p* = 0.023), and risk score (HR =1.732, 95% CI =1.262–2.377, *p* = 0.000) were significantly correlated with OS (**Figure 4B**). However, there was no significant correlation between M, N, gender, and OS. Multivariate Cox regression analysis showed that only the age (HR = 1.029, 95% CI =1.008–1.051, *p* = 0.007) and risk score (HR =1.763, 95% CI =1.285–2.420, *p* < 0.001) could be used as independent prognostic factors for LUSC (**Figure 4C**). These results indicated that the prognosis-related risk values of m5C regulators have the potential to predict prognosis in LUSC patients.

### Expression Levels of *NSUN3* and *NSUN4* in LUSC Patients and Prognostic Analysis

**Figure 5:**
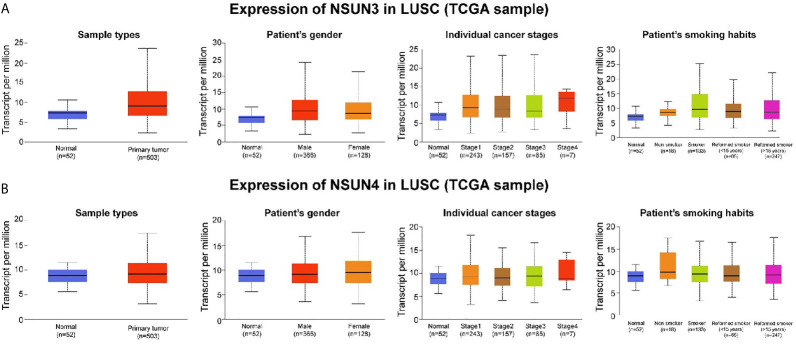
Association between *NSUN3* and *NSUN4* expression and clinicopathological parameters in patients with LUSC (UALCAN). **(A)** Expression of *NSUN3* in normal and LUSC tissues based on sample types, patients’ gender, cancer stage, and smoking habits. **(B)** Expression of *NSUN4* in normal and LUSC tissues based on sample types, patients’ gender, cancer stage, and smoking habits.

To further analyze *NSUN3* and *NSUN4* expression between LUSC tissues and normal lung tissues, we explored their expression in the UALCAN databases. As shown in **Figure 5A** and **Supplementary Figure 2A**, compared with normal tissues (N = 52), the expression of *NSUN3* in LUSC tissues (N = 503) was significantly up-regulated (*p* < 0.05). With respect to gender, the expression of *NSUN3* in men was higher than that in women (*p* < 0.05). In terms of stage, the expression of *NSUN3* was higher in stages I–IV than in normal tissues, and there was statistical significance in stages I–III compared to the normal tissues (*p* < 0.05). However, stage IV was not statistically significant, possibly because the sample size was too small (N = 7), leading to a potential bias. For *TP53* mutation, compared with *TP53* wild patients, the expression level of *NSUN3* in the *TP53* mutant was higher. However, with respect to race, smoking history, and survival, there was no significant difference in the expression of *NSUN3* among the different groups. As shown in **Figure 5B** and **Supplementary Figure 2B**, *NSUN4* expression was significantly up-regulated in LUSC (N = 503) compared with normal tissue (N = 52) (*p* < 0.05). In terms of gender, the expression level of *NSUN4* was higher in females, but this difference was not statistically significant. With regard to stage, the expression level of *NSUN4* in stage I–III cancer tissues was higher than that in normal tissues, but there was no significant difference. According to the survival analysis curve, the OS in patients with high *NSUN4* expression was significantly shorter than that in patients with low *NSUN4* expression (*p* < 0.05). It may be due to the uneven distribution of sample size, but there were no significant differences in the expression of *NSUN4* in different races, smoking, or *TP53* mutations. These results suggested that *NSUN3* and *NSUN4* were closely related to clinicopathological features and may be the oncogenes in LUSC.

### Protein Expression Difference, Gene Alteration Types and Enrichment Analysis of *NSUN3* and *NSUN4*

**Figure 6:**
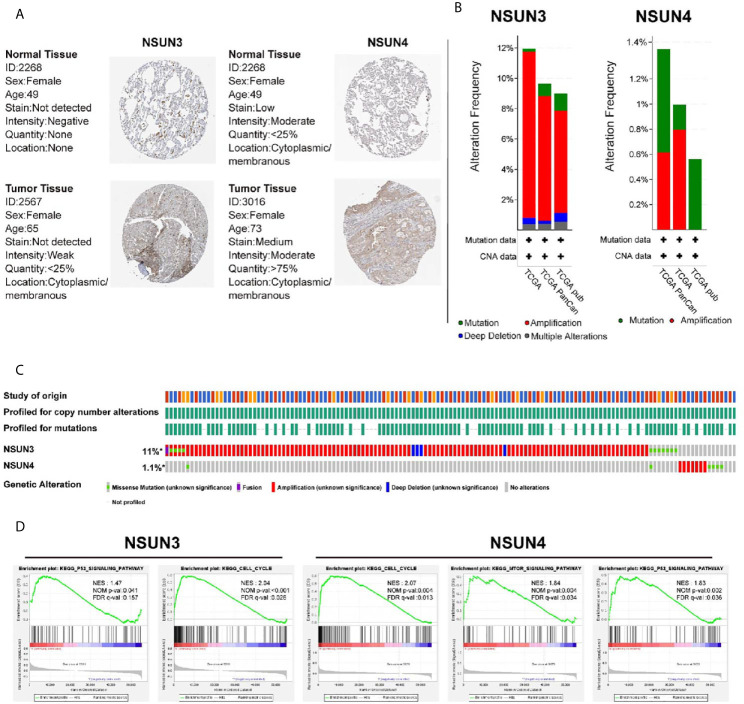
Immunohistochemical analysis (HPA), change frequency analysis (cbiopotal), and gene set enrichment analysis (GSEA) of *NSUN3* and *NSUN4* in LUSC. **(A)** The protein expressions of *NSUN3* and *NSUN4* in LUSC tissues and normal tissues using the HPA. **(B, C)** The frequency of gene changes in *NSUN3* and *NSUN4* in LUSC in three independent studies. **(D)** Enrichment pathway of *NSUN3* and *NSUN4* high expression group in C2KEGG. NES, normalized enrichment score; NOM, nominal; FDR, false discovery rate. Gene sets with NOM *p*-val < 0.05 and FDR *q*-val < 0.25 are considered as significant.

In addition, we attempted to detect the expression levels of *NSUN3* and *NSUN4* in LUSC using the HPA database. IHC detection showed (**Figure 6A**) that *NSUN3* was not present in normal tissues, but low levels of expression (as assessed *via* staining intensity) were observed in cancer tissues with no significant difference between the two; *NSUN4* protein was not expressed in normal lung tissues but was moderately expressed in LUSC tissues. Using the cbiopotals database, we found that in the TCGA database of 1,176 samples, *NSUN3* and *NSUN4* gene changes included missense mutation, fusion, amplification, and deep deletion. The mutation frequencies in *NSUN3* and *NSUN4* were 11 and 1.1%, respectively. *NSUN3* mainly proliferates in TCGA, TCGApan, and TCGA pub data; NSUN4 mainly proliferates in TCGA, TCGApan, and mainly mutation in TCGA pub data (**Figure 6B, C**).To identify the signaling pathways activated by the differential expression of *NSUN3* and *NSUN4* in LUSC, GSEA was performed. Single-gene GSEA analysis showed that the high expression of *NSUN3* was correlated with the *p53* signaling pathway (NSE = 1.47, *p* < 0.05) and cell cycle signaling pathway (NSE = 2.04, *p* < 0.001). Meanwhile, the high expression of *NSUN4* was correlated with the cell cycle signaling pathway (NSE = 2.07, *p* < 0.001), *mTOR* signaling pathway (NSE = 1.84, *p* < 0.001) and *p53* signaling pathway (NSE = 1.83, *p* < 0.001) (**Figure 6D**).

### Relationship Between m5C Regulators and Tumor Immune Microenvironment in LUSC

**Figure 7:**
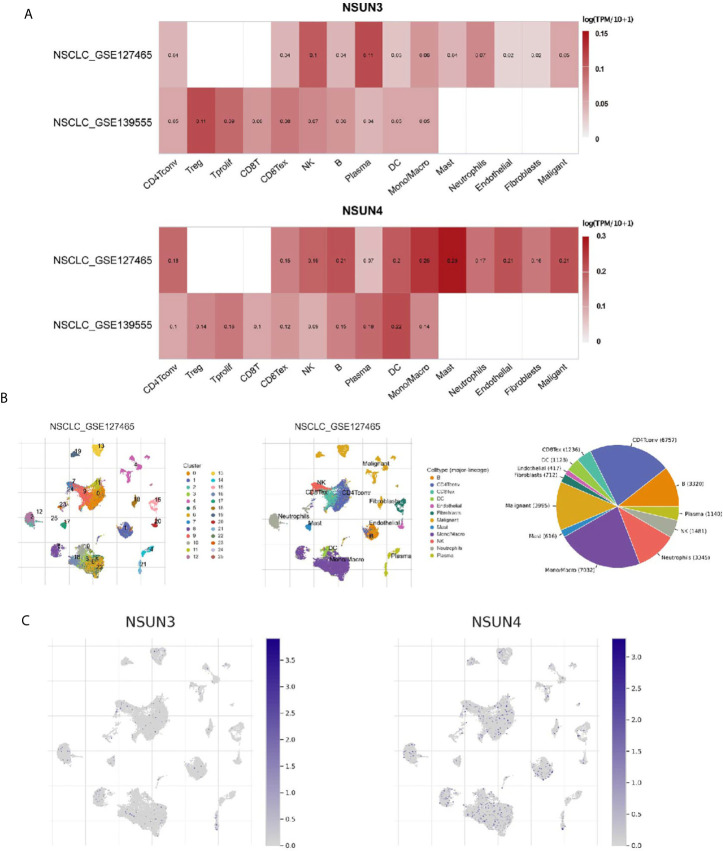
Expression of m5C regulators in tumor microenvironment-related cells (TISCH). **(A)** The expression levels of *NSUN3* and *NSUN4* in the tumor microenvironment-related cells of lung squamous cell carcinoma in the GEO dataset. **(B)** Annotation of all cell types in GSE127465 and the percentage of each type of cell. **(C)** The proportion of *NSUN3* and *NSUN4* in GSE127465 in various types of cells.

The tumor microenvironment (TME), including immune cells, inflammatory cells, and stromal cells (**Table 4**), played an important role in tumor genesis, development, metastasis, recurrence, and drug resistance. Therefore, we used the TISCH database to analyze the degree of invasion of the risk-related genes *NSUN3* and *NSUN4* in TME-related cells. The 18 tumor tissues of GSE124765 contain six LUSC tissues, and the six tumor tissues of GSE139555 contain two LUSC tissues. Immune cells such as neutrophils, macrophages, dendritic cells (DCs), and Tregs had low to moderate infiltration. *NSUN3* had the highest infiltration level in Tregs, and *NSUN4* had the highest infiltration degree in mast cells, followed by monocyte/macrophagecells (**Figure 7A**). As can be seen from the figures, the infiltration degree of *NSUN4* in immune cells was higher than that of *NSUN3*. Using the TISCH database, GSE124765 was divided into 25 cell clusters and 12 types of cells; the pie chart (**Figure 7B**) showed the number of cells in each cell type. The distribution and number of various TME-related cells can be visually observed. The GSE124765 dataset had the largest number of mononuclear macrophages (7,032), followed by CD4+ T cells (6,757). In the database, *NSUN3* was the top gene in plasma cells of the GSE124765 dataset (p < 0.001). It can be seen from **Figure 7C** that *NSUN4* had a higher degree of infiltration in TME-related cells than *NSUN3*, which was consistent with the results shown in **Figure 7A**. In short, the above results indicated that m5C regulators were related to LUSC, especially immune cells.

**Table 4:** The tumor microenvironment includes immune cells/inflammatory cells, stromal cells, and malignant cells.

Immune cells	Conventional CD4 T Cells (CD4Tconv)
	Regulatory T Cells (Treg)
	Proliferative T cells (Tprolif)
	CD8 T Cells (CD8+ T)
	Exhausted CD8 T Cells (CD8Tex)
	Natural Killer Cells (NK)
	B Cells (B)
	Plasma
	Dendritic Cells (DC)
	Monocytes or Macrophages (Mono/Macro)
	Mast Cells (Mast)
	Neutrophils
Stromal cells	Endothelial Cells (Endothelial)
	Fibroblasts (Fibroblasts)
Malignant cells	Malignant Cells (Malignant)

### Immune Cell Infiltration Analysis Showed That the Expression of m5C Regulators Were Correlated With Immune Cells

**Figure 8:**
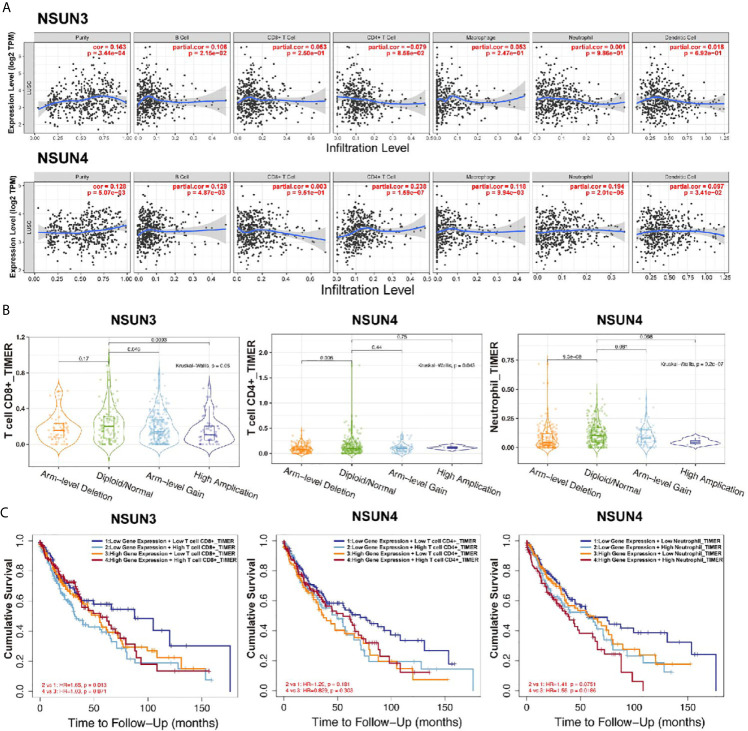
Correlation analysis (TIMER) between m5CRNA methylation regulators and the infiltration levels of the sixmajor immune cells. **(A)** Correlation analysis of the infiltration levels of *NSUN3* and *NSUN4* and the six major immune cells after adjusting for purity. **(B)** Correlation analysis between somatic copy number alterations of *NSUN3* and *NSUN4* and the level of immune cell infiltration. **(C)** Survival analysis of combined *NSUN3* and *NSUN4* expression levels and immune cell infiltration levels.

In order to further analyze the correlation between m5C regulators and immune cells, we used the TIMER database to analyze the correlation between *NSUN3 and NSUN4* and the degree of infiltration of six immune cells (**Figure 8A**). Interestingly, except for the negative correlation between *NSUN3* and the degree of CD4+ T cell infiltration, *NSUN3* and *NSUN4* were positively correlated with the degree of infiltration of most immune cells, and the expression levels of two genes were positively correlated with tumor purity (*p* < 0.05). We then analyzed the relationship between *NSUN3* and *NSUN4* somatic cell copy number variation and the degree of infiltration of the six immune cells (**Figure 8B**). Compared with normal *NSUN3* somatic cells, the expression of CD8+ T cells in each mutant group was down-regulated, and in the arm level gain and high amplification mutation group, the expression of CD8+ T cells was significantly down-regulated (*p* < 0.05). Compared with normal NSUN4 somatic cells, the expression of neutrophils in each mutation group was down-regulated, and the expression of neutrophils in the arm level deletion group was significantly down-regulated (*p* < 0.05), and the expression of CD4+ T cells in the arm level deletion group was significantly down-regulated (*p* < 0.05). Next, we combined m5C regulators with immune cells for survival analysis (**Figure 8C**) and found that in the low *NSUN3* expression group, the survival period of CD8+ T cell infiltration degree was shorter than that in the CD8+T cell infiltration degree group (*p* < 0.05), while the combined analysis of the *NSUN3* high expression group and CD8+ T cell infiltration degree showed no significant difference in survival between the groups. In the *NSUN4* high expression group, the survival period of neutrophils with a high degree of infiltration was shorter than that of neutrophils with a low degree of infiltration (*p* < 0.05), and the combined analysis of *NUSN4* expression level and CD4+T cell infiltration degree showed no significant difference in survival. Therefore, it can be seen from the above analysis that in LUSC, *NSUN3* was closely related to CD8+ T cells, and *NSUN4* was closely related to neutrophils.

## Discussion

More than 150 modifications of RNA have been found, of which m6A, m5C, and m1A are the main ones. They play an important role in regulating gene expression and cell fate. Abnormalities in RNA modification lead to the occurrence of a series of diseases, including cancer (20).Liu et al. found that m6A regulators were significantly differently expressed in LUSC, and METTLE3 and HNRNPC were significantly related to the prognosis of LUSC (8). In this study, we found that m1A and m5C regulators were significantly differently expressed in LUSC. The m5C regulators *NSUN3* and *NSUN4* were highly expressed in LUSC and significantly related to its prognosis. We then used *NSUN3* and *NSUN4* data and constructed a prognostic risk model and used it to divide the patients into high-risk and low-risk groups. We also analyzed the relationship between m5C regulators and the TIM. As far as we know, this is the first in-depth analysis of the role of m5C regulators in LUSC and the first discovery that m5C regulators are associated with tumor immune infiltration.

N1-methyladenosine (m1A) is an important post-transcriptional modification of RNA that was first documented more than 50 years ago (21). Zhao et al. found that, in gastrointestinal cancer, the highly expressed m1A regulatory factor ALKBH3 was associated with poor prognosis and metastasis. After knocking down *ALKBH3*, the expressions of *ErbB*, *mTOR*, and *AKT1S1*, the hub genes of the *ErbB* and *mTOR* pathways, were down-regulated, indicating that *ALKBH3* was related to the mTOR pathway and had an adverse effect on the prognosis of gastrointestinal cancer (22).Studies have shown that the m1A demethylase *ALKBH3* can act on the 5’UTR near the initial translation site of the cytokine macrophage colony stimulating factor (CSF-1) to promote the invasion of breast cancer and ovarian cancer cells, indicating that the regulation of m1A RNA methylation can lead to functional changes in cancer cells. In this study, the expression of m1A regulators was significantly different between LUSC tissues and adjacent normal tissues. However, in the univariate Cox regression analysis, the m1A regulators had no significant correlation with prognosis, which may be due to inherent biases in TCGA database, the nature of LUSC, and the normal sample size distribution, all of which could have caused bias; furthermore, this study is the first one to assess the relationship between m1A regulators and LUSC, and more *in vivo* and *in vitro* studies are needed for further verification.

m5C regulators are closely related to cell growth and development. Previous studies (23, 24) have reported that the m5C regulators *NSUN2* was the downstream target gene of the oncogene *MYC*. Upregulation of *MYC* promotes cell cycle progression and upregulation of *NSUN2*, with the highest expression observed in the S phase. In breast cancer, the expression of *NSUN2*was significantly associated with the clinical stage, tumor type, pathological differentiation, estrogen receptor, progesterone receptor, and Ki-67 expression levels (24). This shows that *NSUN2* is a powerful and clinically significant biomarker in breast cancer and can be used as a potential therapeutic target for breast cancer. In NSCLC, *NSUN1* has been identified as a prognostic marker (25),but it was done mainly in the context of the LUAD research. In this study, m5A regulators were significantly differentially expressed between LUSC and normal tissues. The m5C regulators *NSUN3* and *NSUN4* were significantly correlated with prognosis as risk factors, and these two genes were used to construct a prognostic risk model. The survival rate of the low-risk group was higher (*p* < 0.05), thus indicating the use of this risk value as an independent prognostic factor. This study is the first in-depth study on the m5C regulators in LUSC, and the results need to be further verified in *in vitro* and *in vivo* studies. *NSUN3* is required for the deposition of m5C on the anticodon loop of the mitochondrial transfer RNA methionine (mt-tRNAMet). The mutation of m5C in mt-tRNAMet results in a lack of 5-formylcytosine (fC) at the same tRNA position, indicating that *NSUN3* is required for efficient mitochondrial translation (26). *NSUN4*, which forms a complex with MTERF4, is necessary for mitochondrial ribosome biogenesis. Mitochondrial translation is disrupted after gene knockout in *NSUN4*-deficient mice (27), and research on *NSUN3* and *NSUN4* in cancer is limited.

In this study, we found that the mRNA expression level of *NSUN3* in LUSC tissues was significantly upregulated compared with that in normal tissues and was closely related to clinicopathological features. However, no significant difference in IHC was found in HPA data, suggesting that *NSUN3* itself may regulate its protein expression through post-transcriptional modification. The mRNA expression level of *NSUN4* in LUSC tissues was significantly upregulated compared with that in normal tissues, the expression level of *NSUN4* was higher in women, and the OS in patients with high *NSUN4* expression was shorter. However, compared with *NSUN3*, the differences in clinicopathological features of *NSUN4* were not significant, which may be related to the differences in the number of TCGA LUSC tissues and normal tissues. Previous studies have also shown that *NSUN3* mutations affected mitochondrial translation. This study found that 11% of LUSC tissues had mutations in *NSUN3* while only 1.1% of LUSC tissues had mutations in *NSUN4*. These mutations included missense mutation, fusion, amplification, and deep deletion, and mainly affected proliferation. Gene mutations can cause phenotypic changes and are important pathogenic factors for tumors. Therefore, the analysis of m5C regulators mutation plays an important role in understanding the occurrence and development of LUSC and has therapeutic guiding significance.

Through GSEA, we found that the up-regulation of *NSUN3* and *NSUN4* were closely related to the *p53* signaling pathway, cell cycle signaling pathway, and *mTOR* signaling pathway.*p53* is a tumor suppressor gene, which plays a major role in inhibiting tumor angiogenesis (28). *p53* can maintain the cell cycle at the G1/S regulatory point, thereby activating DNA repair proteins, initiating apoptosis, or inducing growth stagnation (29). *p53* mutations occur in approximately 50% of NSCLC cases (30). In addition to the loss of tumor suppressor function, *p53* mutation can also promote malignant progression and enhance cell invasion and metastasis (31, 32). Studies have shown that *p53* mutations had a synergistic effect with the oncogene Kras, which could shorten the incubation period of LUAD and increase the ability of metastasis, while somatic mutations in *p53* are the most common co-mutations in *EGFR*-mutated LUAD (54.6−64.5%) (33). The *p53* mutation is related to the increased expression of PD-L1 in tumor cells in the inflammatory tumor immune microenvironment and *KRAS* mutation in NSCLC. Some of these events could be due to the activation of the nuclear factor kB (NF-*κ*B) pathway due to mutations in *p53*, leading to enhanced cellular immunogenicity (34, 35). Previous studies have reported that cell cycle-related proteins are closely related to tumor progression in NSCLC (36, 37). The rapamycin (*mTOR*) signaling pathway is involved in various cell functions. Rapamycin (*mTOR*) is a serine/threonine kinase that regulates cell growth, survival, metabolism, autophagy and senescence. Dysregulation of the *mTOR* pathway is more common in squamous cell lung cancer than in adenocarcinoma, and patients with mutant *EGFR* always show abnormal PI3K/AKT/mTOR activation, which leads to resistance to clinical treatment with EGFR-tyrosine kinase inhibitor (EGFR-TKI) (38). However, the correlation between *NSUN3* and *NSUN4* in LUSC and the *p53* signaling pathway, cell cycle signaling pathway, and *mTOR* signaling pathway has been reported for the first time, and its regulatory mechanism needs to be further clarified.

In recent years, the TIM has received extensive attention in tumor research. The tumor immune escape mechanism is an early event of malignant precancerous lesions progressing to invasive cancer (39). In NSCLC, LUSC has a higher degree of tumor-related neutrophil infiltration than LUAD. Neutrophils are immunosuppressive factors, and the degree of infiltration is inversely proportional to the degree of CD8+ Tcell infiltration (40). Jiang et al. (41) performed whole-exome sequencing of 189 cases of surgically resected LUSC and found that tumors with mutations in *KEAP1* or *NFE2L2* had a higher level of oxidative stress, which may cause CD8+ tumor-infiltrating lymphocytes and other immune cells to be destroyed and DNA damage levels to be increased, leading to an increase in somatic mutations in tumor cells. Rizvi et al. (42) also found that in NSCLC patients treated with anti-programmed cell death (PD)-1and anti-programmed death-Lig and 1 (PD-L1), TMB was not associated with PD-L1 expression. In this study, we used the TISCH and TIMER databases to analyze the correlation between m5A regulators in LUSC and the six major immune cells in the tumor immune microenvironment, and found that *NSUN3* and *NSUN4* were expressed to a certain extent in immune cells. *NSUN4* was stronger than *NSUN3*, but both had a certain correlation with the six major immune cells. Furthermore, *NSUN4* had the strongest correlation with CD4+T cells and tumor-associated neutrophils, which is consistent with the results shown in previous studies. The m5A regulators were related to the TIM, but the specific regulatory mechanism needs further study.

However, there are still some shortcomings in this study. First, there were fewer studies on LUSC compared to LUAD. The uneven distribution of LUSC samples (N = 503) and normal tissue samples (N = 52) in the TCGA database resulted in subsequent impacts on the results related to m5A regulators and clinical pathology. Second, we did not find a large sample of data in the GEO database. The two data sets GSE3349 and GSE19188 used in this study had a small sample size and it had certain limitations. Third, predicted differentially expressed genes and prognostic risk score models failed to be validated *in vitro* and *in vivo*. Fourth, although the TIMER database (2.0) can perform correlation analysis on differential genes and immune cells in cancer, it failed to correlate clinicopathological features with the degree of immune cell infiltration. Therefore, more in-depth research is needed to overcome these problems.

In short, the current research on LUSC is far behind that on LUAD. Our research showed that there were significant differences in the expression of m5C and m1A regulators in LUSC and adjacent tissues, and we have developed prognostic risk markers using m5C regulators and found that m5C regulators could affect the TIM. Therefore, m5C regulators are expected to become prognostic markers in LUSC and provide strategies for the treatment of this disease.

## Conclusion

In summary, we found that m5C regulators could predict the clinical prognostic risk in LUSC patients and regulate the TIM, thus possessing the potential to become new prognostic indicators in LUSC patients.

## Data Availability Statement

The datasets presented in this study can be found in online repositories. The names of the repository/repositories and accession number(s) can be found in the article/**Supplementary Material**.

## Author Contributions

JP and YX designed this experiment, JP and ZH were responsible for literature review, data collection, analysis and writing, and YX was responsible for modification. All authors contributed to this article and approved the submitted version.

## Conflict of Interest

The authors declare that the research was conducted in the absence of any commercial or financial relationships that could be construed as a potential conflict of interest.
