# Supplementary material for: m5C RNA Methylation Regulators Predict Prognosis and Regulate the Immune Microenvironment in Lung Squamous Cell Carcinoma
Source: Front Oncol. 2021 Jun 9;11:657466. doi: 10.3389/fonc.2021.657466 (PMC8237756; doi:10.3389/fonc.2021.657466)
Supplement: Supplementary file 1 [file DataSheet_1.docx]

Supplementary Material


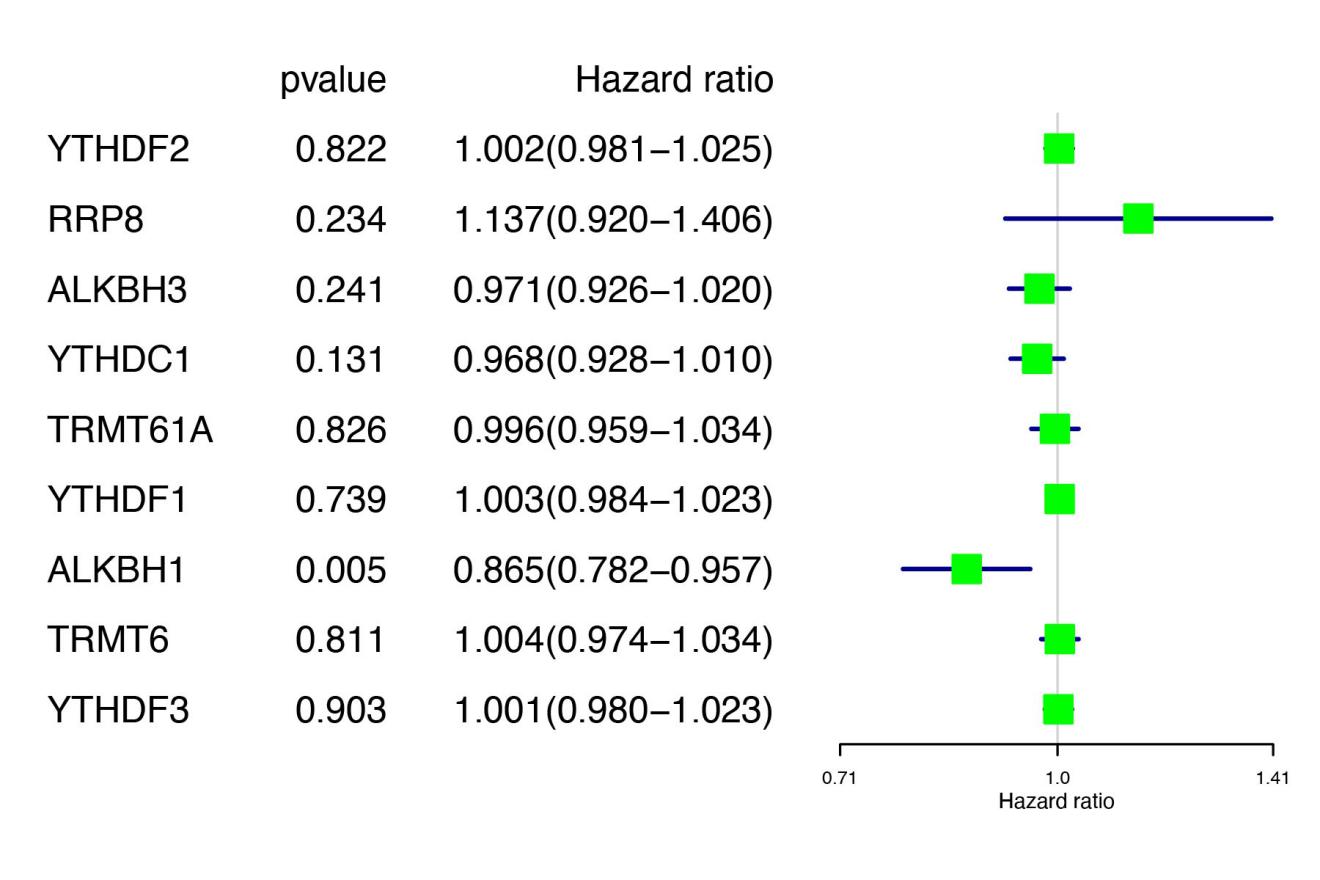


**Supplementary Figure 1.**Univariate analysis of the m1A regulators.


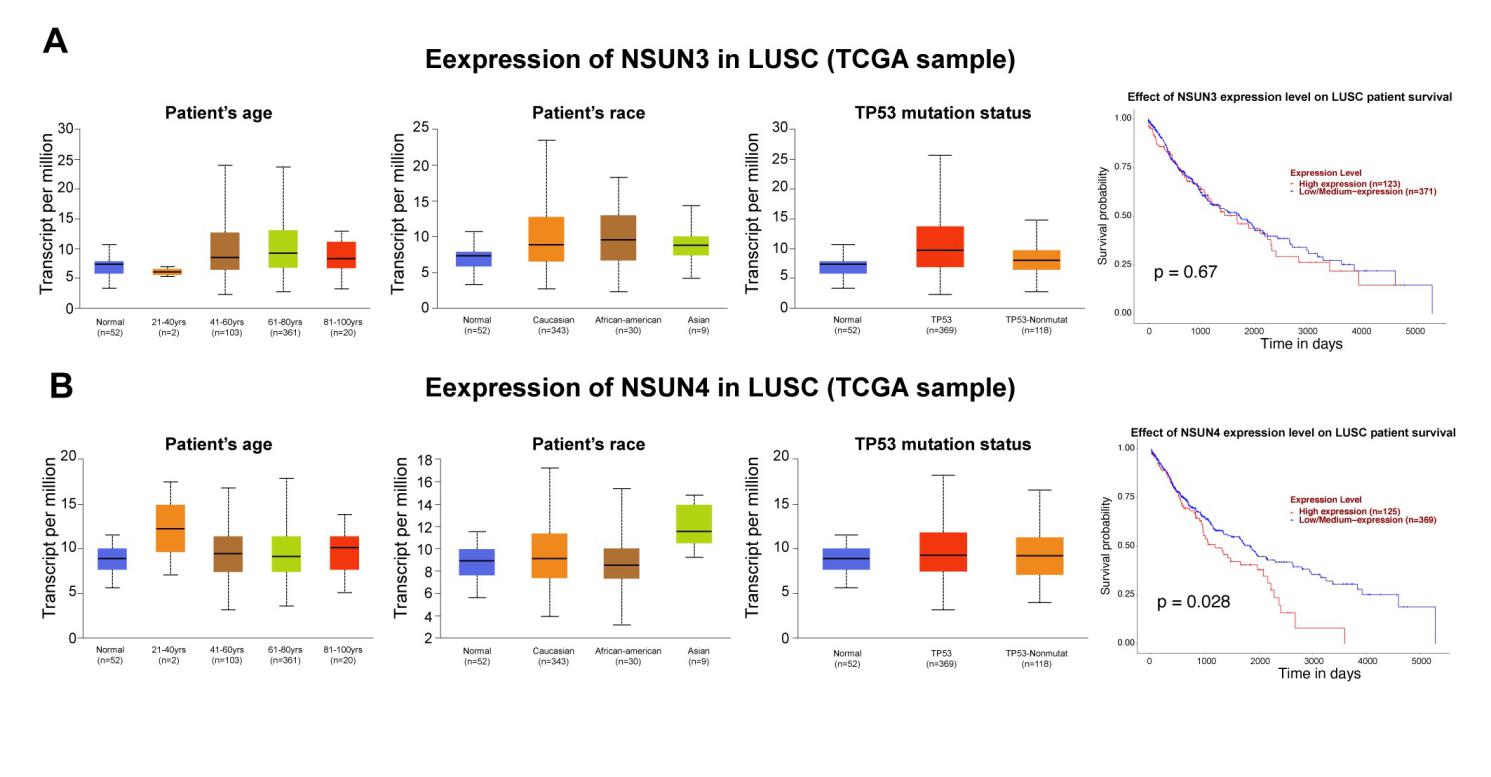


**Supplementary Figure 2.** Association between *NSUN3* and *NSUN4* expression and clinicopathological parameters in patients with LUSC (UALCAN). **(A)** Expression of *NSUN3* in normal and LUSC tissues based on patients’age, patients’race, TP53 mutation status and the effect of *NSUN3* expression level on LUSC patient survival. **(B)**Expression of *NSUN4* in normal and LUSC tissues based on patients’age, patients’race, TP53 mutation status and the effect of *NSUN4* expression level on LUSC patient survival.

| m5C regulators | Expression level | | Log_2_FC | *P*-velue |
| --- | --- | --- | --- | --- |
|  | Control | Lung squamous cell carcinoma |  |  |
| NSUN6 | 2.814363978 | 1.890451627 | 0.574077989 | 2.21E-09 |
| NSUN5 | 9.864093955 | 4.445676881 | 1.149783446 | 4.76E-22 |
| ALYREF | 61.61679417 | 21.84867147 | 1.495778065 | 2.14E-28 |
| DNMT1 | 13.53670915 | 6.223152033 | 1.121159657 | 4.39E-19 |
| DNMT3B | 2.626512853 | 0.369784646 | 2.828391415 | 2.48E-28 |
| TET2 | 2.260273452 | 1.952009713 | 0.211537092 | 0.108861824 |
| NSUN2 | 25.62473515 | 9.365234208 | 1.452150114 | 1.52E-27 |
| TRDMT1 | 0.553026007 | 0.806736038 | -0.544749377 | 2.98E-12 |
| NSUN7 | 1.212726379 | 1.664010999 | -0.45641089 | 5.84E-05 |
| DNMT3A | 5.31402955 | 2.228390934 | 1.253803899 | 2.29E-21 |
| YBX1 | 306.4194176 | 191.2191892 | 0.680280415 | 2.19E-14 |
| NSUN4 | 3.472517601 | 3.014368051 | 0.204126428 | 0.009378237 |
| NSUN3 | 2.813874394 | 1.774022785 | 0.665533391 | 1.71E-05 |
| m1A regulators | Expression level | | Log_2_FC | *P*-velue |
|  | Control | Lung squamous cell carcinoma |  |  |
| YTHDF2 | 20.06925851 | 22.70893909 | 0.178272993 | 0.015230462 |
| RRP8 | 2.520220612 | 2.487262063 | -0.018991507 | 0.231224929 |
| ALKBH3 | 6.590550061 | 8.464055095 | 0.360950138 | 1.30E-06 |
| YTHDC1 | 10.31424837 | 11.25122384 | 0.125443246 | 0.136582582 |
| TRMT61A | 5.066602347 | 8.162574477 | 0.688005647 | 4.82E-11 |
| YTHDF1 | 16.33556984 | 24.86300239 | 0.605983741 | 2.22E-19 |
| ALKBH1 | 3.429169224 | 4.373833307 | 0.351039137 | 2.34E-05 |
| TRMT6 | 4.327496857 | 10.6748966 | 1.302617418 | 2.03E-26 |
| YTHDF3 | 12.41726282 | 13.73406175 | 0.145411165 | 0.113716289 |

**Supplementary table 1.** Differentially expressed genes of m5C regulators and m1A regulators in TCGA database.

| m5C regulators | Expression level | | Log_2_FC | *P*-velue |
| --- | --- | --- | --- | --- |
|  | Control | Lung squamous cell carcinoma |  |  |
| NSUN6 | 0.718730601813954 | 0.47312067 | -0.603242922224121 | 0.003521824 |
| NSUN5 | 0.441173229 | 0.884361942 | 1.003291693 | 5.46E-08 |
| DNMT1 | 0.3210398 | 1.348059147 | 2.070059731 | 2.29E-12 |
| DNMT3B | 0.240107082 | 1.975161041 | 3.040220426 | 1.53E-11 |
| TET2 | 0.594225406 | 0.529351069 | -0.166785446 | 0.252692693 |
| NSUN2 | 0.362320102 | 1.087323095 | 1.58544394 | 1.08E-10 |
| TRDMT1 | 0.692257856 | 0.440014016 | -0.653760042 | 2.32E-05 |
| NSUN7 | 1.251178417 | 0.465565566 | -1.426231268 | 0.003953887 |
| DNMT3A | 0.53057849 | 0.80960557 | 0.609653025 | 0.00020959 |
| YBX1 | 0.471794345 | 0.898300462 | 0.929039949 | 5.08E-06 |
| NSUN4 | 0.491446849 | 0.691430759 | 0.492549388 | 0.000750519 |
| NSUN3 | 0.504331081 | 0.73954819 | 0.552273019 | 0.003011694 |

**Supplementary table 2.** Differentially expressed genes of m5C regulators in GEO database
